# Predictors of Chronic Fatigue Syndrome and Mood Disturbance After Acute Infection

**DOI:** 10.3389/fneur.2022.935442

**Published:** 2022-07-25

**Authors:** Carolina X. Sandler, Erin Cvejic, Braulio M. Valencia, Hui Li, Ian B. Hickie, Andrew R. Lloyd

**Affiliations:** ^1^Laboratory Viral Immunology Systems Program, Kirby Institute, The University of New South Wales (UNSW Sydney), Sydney, NSW, Australia; ^2^Sport and Exercise Science, School of Health Science, Western Sydney University, Sydney, NSW, Australia; ^3^Menzies Health Institute Queensland, Griffith University Brisbane, Queensland, QLD, Australia; ^4^The University of Sydney, Faculty of Medicine and Health, School of Public Health, Sydney, NSW, Australia; ^5^The University of Sydney, Brain and Mind Centre, Sydney, NSW, Australia

**Keywords:** acute infection, chronic fatigue, mood disturbance, genetics, post-infective syndrome, predictors

## Abstract

Prospective cohort studies following individuals from acute infections have documented a prevalent post-infective fatigue state meeting diagnostic criteria for chronic fatigue syndrome (CFS) – that is, a post-infective fatigue syndrome (PIFS). The Dubbo Infection Outcomes Study (DIOS) was a prospective cohort following individuals from acute infection with Epstein-Barr virus (EBV), Ross River virus (RRV), or Q fever through to assessment of caseness for CFS designated by physician and psychiatrist assessments at 6 months. Previous studies in DIOS have revealed that functional genetic polymorphisms in both immunological (pro- and anti-inflammatory cytokines) and neurological (the purinergic receptor, P2X7) genes are associated with both the severity of the acute infection and subsequent prolonged illness. Principal components analysis was applied to self-report data from DIOS to describe the severity and course of both the overall illness and concurrent mood disturbance. Associations between demographics and acute infection characteristics, with prolonged illness course as well as the PIFS outcome were examined using multivariable statistics. Genetic haplotype-driven functional variations in the neuropeptide Y (NPY) gene previously shown to be associated with brain responses to stress, and to trait anxiety were also examined as predictors. The sample included 484 subjects (51% female, median age 32, IQR 19–44), of whom 90 (19%) met diagnostic criteria for CFS at 6 months. Participants with greater overall illness severity and concurrent mood disturbance in the acute illness had a more prolonged illness severity (HR = 0.39, 95% CI: 0.34–0.46, *p* < 0.001) and mood disturbance (HR = 0.36, 95% CI: 0.30–0.42, *p* < 0.001), respectively. Baseline illness severity and RRV infection were associated with delayed recovery. Female gender and mood disturbance in the acute illness were associated with prolonged mood disturbance. Logistic regression showed that the odds of an individual being diagnosed with PIFS increased with greater baseline illness severity (OR = 2.24, 95% CI: 1.71–2.94, *p* < 0.001). There was no association between the NPY haplotypes with overall illness severity or mood disturbance either during the acute illness phase or with prolonged illness (*p* > 0.05). Severe acute infective illnesses predicted prolonged illness, prolonged mood disturbance and PIFS. These factors may facilitate early intervention to manage both PIFS and mood disturbances.

## Introduction

Acute infections are a universal experience and are a significant cause of morbidity, mortality, and economic burden globally. Prospective cohort studies following individuals from acute infection with both viral and non-viral pathogens have documented a prevalent post-infective fatigue state meeting diagnostic criteria for chronic fatigue syndrome (CFS) – that is, post-infective fatigue syndrome (PIFS) (1–3). These studies include the Dubbo Infection Outcomes Study (DIOS) which is the basis of this report, as well several other prospective cohort studies following individuals from infection including Epstein-Barr virus (EBV) and other pathogens, including COVID-19 (1–3). By contrast, a well-controlled longitudinal study in general practice in the United Kingdom found that patients presenting with minor symptomatic infections, such as common colds or gastroenteritis did not experience post-infective fatigue (4). Studies of PIFS offer the opportunity to better understand CFS as: the syndrome is relatively homogeneous; the population can be studied in evolution, including prior to the onset of CFS; and the cohorts incorporate closely matched control subjects and biological samples (i.e., those of comparable demographics who recover promptly from the same infection).

The physiological (e.g., fever), behavioral (e.g., fatigue, hypersomnia, pain), and psychological (e.g., cognitive impairment, mood disturbance) manifestations of the acute sickness response to infection are driven by the host response to the pathogen and are stereotyped across many viral and non-viral infections (5). These central nervous system (CNS)-mediated changes are neurobehavioural responses to the acute infection that work in conjunction with the host immune response to facilitate pathogen clearance and recovery (5). The physical (e.g., fatigue) and psychological symptoms (e.g., mood) are thought of as having a “protective role”, so that the goal of these symptoms are to preserve energy to combat the pathogen during the acute illness. Persistence of these symptoms for weeks or months beyond the acute febrile phase of infection is common (2, 3), resulting in negative impacts on functional status and quality of life. Findings from prospective, retrospective, and experimental studies have shown associations between acute infection and subsequent mood disturbance, notably major depression (6). The available evidence suggests that genetic, neuroendocrine, autonomic and psychosocial factors may interact to increase the likelihood of severe and prolonged mood disturbance in the context of acute infections (6).

Numerous prospective cohort studies have examined biological, psychological, and social factors as predictors of delayed recovery or PIFS following an acute infection (3). The most consistently reported predictor of PIFS has been features associated with more severe acute illness, including self-reported symptom severity, poor functional status, and the presence of biochemical hepatitis in acute EBV infection (1, 7, 8). There is also some evidence for self-reported anxiety, perceived stress, neuroticism, negative beliefs about the acute illness, as well as pre-morbid distress as risk factors for prolonged recovery (7). It should be noted that the only prospective cohort study which collected data *prior* to the acute illness found that pre-morbid mental health and personality characteristics did not predict PIFS following EBV (8). Female gender has also been identified as a risk factor for developing chronic fatigue as a symptom (defined as persistent fatigue at 6 months but not applying the CFS diagnostic criteria) (9–12). In the COVID-19 context, a systematic review found that illness severity during the acute illness (represented by hospitalization, intensive care unit admission, duration of hospital stay) was associated with the symptom report of prolonged fatigue at 12 weeks follow up (3). A recently published prospective cohort study of 303 subjects with COVID-19 showed that severe illness and low mood at baseline were associated with persistent fatigue at 6 months (13).

The pattern and relative severity of the broad symptom domains within the acute sickness response and the prolonged illness complex, such as fatigue, pain, and mood disturbance, are stable over time (14), suggesting a shared pathophysiology between the acute sickness response to infection and PIFS. The association of genetic factors with both acute-phase symptom severity and prolonged symptoms have been documented by our group (14–18). Previous studies in DIOS have revealed that functional genetic polymorphisms in both immunological (pro- and anti-inflammatory cytokines) and neurological (the purinergic receptor, P2X7) genes are associated with both the severity of the acute infection and subsequent prolonged illness.

Another potential candidate for the effect of genetic polymorphisms is neuropeptide Y (NPY), which is a neurotransmitter found in both the central and peripheral nervous systems. In the periphery, NPY is released from nerve endings reflecting sympathetic autonomic activity. In the brain, NPY is anxiolytic, inhibitory of sympathetic activity and causes lowering of blood pressure and heart rate. Haplotype-driven functional polymorphisms in the NPY gene have been previously linked to both depression and anxiety; (19–21), and in patients with CFS, plasma levels of NPY have been correlated with stress, depression, and cognitive function. The functional polymorphisms in NPY may therefore predispose individuals to anxiety and low mood, which are recognized features of the acute sickness response to infection and PIFS.

This study aimed to firstly describe the acute phase illness characteristics and the longitudinal illness course; and secondly to examine demographics, baseline illness characteristics, and NPY polymorphisms as predictors of prolonged illness, mood disturbance, and PIFS.

## Materials and Methods

### Study Design and Study Population

DIOS was a large Australian prospective cohort study following individuals from a documented acute infection with Epstein-Barr Virus (EBV: the causative agent of infectious mononucleosis), Ross River virus (RRV: an Australian mosquito-borne pathogen which causes polyarthritis), and Q fever (QF: a zoonotic infection caused by the intracellular bacterium, *Coxiella burnetii*). The DIOS cohort has been previously described in detail (1, 22, 23). Briefly, the study enrolled *n* = 484 Caucasian adults who were provisionally diagnosed in primary care as having EBV, RRV, or QF infection based on screening by pathogen-specific IgM enzyme-linked immunosorbent assays (ELISA). Participants were recruited as soon as possible following the initial illness onset (time since symptom onset: median 30 days; IQR: 19 days). Initial serological diagnoses were confirmed by testing acute and convalescent sera. Subjects for whom the provisional diagnosis could not be verified were included in the analysis but have been classified as serologically “unconfirmed.” All participants were prospectively followed at 2–3 weeks, 4–6 weeks, at three-monthly intervals until recovery. All participants provided informed written consent.

### Self-Report Measures, Clinical Interviews and Case Definition

At baseline, a structured medical history interview was conducted, assessing subject's pre-morbid physical and mental health status. At each assessment time point, self-report questionnaires documenting physical and psychological health were administered, and blood samples were collected. At baseline patients were asked to report on their symptoms “since illness onset.” For this analysis, data from the Somatic and Psychological Health Report (SPHERE) questionnaire (24), the Brief Disability Questionnaire (BDQ) (25), and the Profile of Mood States (POMS) (26) were used. The SPHERE is a 34-item instrument which assesses a range of physical and psychological symptoms and prompts patients to report their symptoms over the “past few weeks.” Responses are scored based on the prevalence of symptoms: “none of the time or some of the time” (scored as 0), “a good part of the time” (scored as 1), and “most of the time” (scored as 2). The BDQ and POMS were used to capture functional impairment and mood disturbance, respectively.

Subjects were classified as a “case,” that is, given a diagnosis of post-infective fatigue syndrome (PIFS), if they met the diagnostic criteria for chronic fatigue syndrome (CFS) (27) at 6 months after the onset of symptoms, following comprehensive medical assessment including laboratory investigations by a specialist physician, and structured mental health assessment by a psychiatrist.

### Endophenotype and Severity Indices

Principal component analysis (PCA) was applied to baseline self-report symptom data from the SPHERE questionnaire (14, 22, 23) to derive “illness severity” and “mood disturbance” principal components (PCs). Participants with a time since symptom onset at baseline of ≤42 days were included in the derivation phase so that the item loadings reflected the acute phase of the illness. Only SPHERE items with report rates ≥ 20% for “a good part of the time” or “most of the time” were considered for inclusion in the overall illness severity PC. A broad array of physical, behavioral, and mood items were candidates for considered inclusion in the mood disturbance PC. Non-salient items with factor loading scores <0.3 were sequentially removed to refine the resulting components. Scores on the BDQ were used as external validation of the anticipated functional impact of higher scores on the illness severity and mood disturbance PCs. The mood disturbance PC was additionally validated against the *depression/dejection, tension/anxiety* and *fatigue/inertia* subscales of the POMS (26).

Component loadings (i.e., the weighting assigned to individual SPHERE item responses) were then applied to the follow-up dataset. On this scale, a value of 0 represents the average PC score for illness severity or mood disturbance during the acute illness, with values measured in standard deviation (SD) units. To describe the illness course in relation to baseline severity, the sample was split into quartiles based on the illness severity and mood disturbance PC scores at baseline, referring to quartile one as “mild,” quartile two as “moderate,” quartile three as “major” and quartile four as “severe.”

### Empirical Definition of Recovery

As both fatigue and mood disturbance are common complaints in primary care, an empirical definition of recovery from prolonged illness was determined using within-sample negative threshold scores. Subjects who responded “no” to the question “do you still have symptoms (of the initial illness)” in data collected at 12 months were considered “recovered.” The 75^th^ percentile of the illness severity and mood disturbance PC scores of this recovered group (*n* = 161) was designated as the recovery threshold (i.e., 75% of recovered participants had PC scores less than or equal to this threshold value).

### Genotyping

Genomic DNA was extracted from stored peripheral blood mononuclear cells using the Wizard DNA kit (Promega, Madison, USA). DNA was quantified using NanoDropR ND-1000 (BioLab, Mulgrave, Australia), and the quality verified by gel electrophoresis. Seven single nucleotide polymorphisms (SNPs) within the neuropeptide Y (NPY) gene, which form the previously characterized functional haplotype block, (28) were genotyped using DNA from DIOS subjects. The SNPs included: rs16139, rs16147, rs16475, rs17149106, rs5573, rs5574, and the insertion/deletion variant rs3037354. Genotyping of selected SNPs was performed using the Agenda Bioscience MassARRAY platform at the Australian Genome Research Facility. Pairwise linkage disequilibrium (LD) was analyzed using Haploview (http://www.broad.mit.edu/mpg/haploview), and PLINK (https://zzz.bwh.harvard.edu/plink/) was used to assign diplotypes to each individual.

### Statistical Analysis

Statistical analyses were performed in IBM SPSS Statistics for Windows, Version 26.0. (Armonk, NY: IBM Corp). Principal component analysis was used to generate the overall illness severity and mood disturbance PCs. Associations between PC scores and subscales of validated questionnaires (BDQ, POMS) were conducted using Spearman's rank-order correlation statistic (ρ). Univariate linear regression models were used to identify correlates of overall illness severity and mood disturbance scores at baseline. Cox-regression was used to analyse the association between baseline illness severity and protracted illness; mood disorder was not included in this analysis as it was co-linear with illness severity. Illness duration was designated as the time (in days) from symptom onset to the midpoint between the last known time point when the individual had not recovered (i.e., component scores greater than the recovery threshold) and the first time point when they had recovered (i.e., component score below the recovery threshold). Individuals who had not recovered by the 6-month assessment were censored at this time point, and individuals who were lost to follow-up before their 6-month assessment were censored at their last known unrecovered time point. Covariates included age, sex, pathogen, baseline illness severity and baseline mood severity. Logistic regression was used to calculate odds ratios of covariates for PIFS outcomes. A *p*-value of 0.05 was considered the threshold for statistical significance.

## Results

### Participant Characteristics

Participant characteristics are presented in Table 1. The sample of 484 subjects (51% female), had a median age of 32 years (IQR 19–44) and a median time since symptoms onset at enrolment of 30 days (IQR 22–41). Of the enrolled subjects, 30% (*n* = 144) had confirmed EBV infection, 20% (*n* = 98) had confirmed RRV infection, 17% (*n* = 84) had confirmed Q fever, and the remaining 33% (*n* = 158) were classified as “unconfirmed” where the pathogen provisionally implicated as causing the acute infectious illness was not serologically confirmed.

**Table 1:** Demographics of the DIOS cohort (*n* = 484) and PIFS sub-group (*n* = 90).

	**Total (*N* = 484)**	**PIFS (*n* = 90)**
Median age (IQR)	32 (19–44)	38 (22–47)
Female; *n* (%)	247 (51)	42 (46)
Median days since symptom onset (IQR)	30 (22–41)	34 (25–47)
Infection type; *n* (%)		
*Epstein-barr virus*	144 (30)	15 (17)
*Ross-river virus*	98 (20)	25 (28)
*Q-fever*	84 (17)	19 (21)
*Unconfirmed*	158 (33)	31 (34)
Median days out of role (IQR) in acute illness by infection type		
*Epstein-barr virus*	14 (6–20)	16 (7–28)
*Ross-river virus*	6 (0–20)	10 (3–22)
*Q-fever*	14 (5–28)	14 (4–28)
*Unconfirmed*	10 (2–18)	10 (2–25)
Median days in bed (IQR) in acute illness by infection type		
*Epstein-barr virus*	7 (1–10)	11 (2–24)
*Ross-river virus*	0 (0–3)	1 (0–4)
*Q-fever*	7 (3–14)	7 (0–14)
*Unconfirmed*	2 (0–7)	4 (0–7)

*IQR, Interquartile range; PIFS, post-infective fatigue syndrome*.

Across all four infection groups at enrolment, the majority of the sample (88%; n=329) reported being “limited in the kind or amount of vigorous activity” they could undertake and were unable to complete their usual daily routine (56%; *n* = 209). Participants had a median of 10 days in the last month (IQR: 3–20) in which they were unable to carry out their usual daily activities fully (days out of role) and spent a median of 3 days in bed due to the acute illness (IQR: 0–8). Mood disturbance also impacted function, with 49% (*n* = 182) of the cohort reporting a “deterioration in social relations with friends, workmates or others.”

Using the empirically derived designation of illness recovery, the median recovery interval was 81 days (IQR 42 – 170), and by 150 days, 60% (*n* = 290) had recovered. At the extreme end of illness duration, 90 patients (19%) were designated as PIFS cases (i.e., meeting diagnostic criteria for CFS) at 6 months. The characteristics of this sub-group are presented in Table 1.

### PC Derivation and Validation

Individual loading scores of the items included in the illness severity and mood disturbance PC scores are provided in Supplementary Tables 1, 2. The overall illness severity PC solution included 19 items (which accounted for 42.1% of the variance) and encompassed physical symptom items (e.g., “Muscle pain after activity?”), neuro-behavioral items (e.g., “Poor concentration?”) as well as mood items (e.g., “Rapidly changing moods?”). The mood disturbance PC included 12 items (which accounted for 48.6% of response variance) related to anxiety (e.g., “Feeling nervous or tense?”) and depression (“Feeling unhappy/depressed?”).

Pairwise correlations were used to functionally validate the PC scores. The BDQ total score (an overall measure of disability) was moderately correlated with the overall illness severity PC score (Spearman's ρ = 0.46, *n* = 362, *p* < 0.01) and the mood disturbance PC score (ρ = 0.41, *n* = 363, *p* < 0.01) in the acute illness. The mood disturbance PC score was strongly correlated with the POMS depression (ρ = 0.55, *n* = 402, *p* < 0.001) and anxiety subscales (ρ = 0.57, *n* = 417, *p* < 0.01). As expected, given that mood disturbance is an intrinsic component of the acute sickness response, and the overlapping items, the baseline illness severity and mood disturbance PC scores were strongly correlated (ρ = 0.84, *n* = 479, *p* < 0.001).

### NPY Genotype in the DIOS Population

All of the NPY SNPs were in Hardy-Weinberg equilibrium in the study population. The linkage disequilibrium between the seven maker SNPs and resulting haplotype blocks are reported in Supplementary Figure 1. The haplotype blocks derived from the DIOS cohort were consistent with the previous report (28), maintaining a similar structure and allele frequency with the exception of the insertion/deletion (rs3037354). There were four haplotypes in the DIOS population with the insertion and deletion variants being merged when compared to the previously reported five functional haplotypes (28).

### Associations With Overall Illness Severity and Mood Disturbance During the Acute Illness Phase

Univariate general linear models (GLM) for illness severity and mood disturbance PC scores are provided in Table 2. Females reported significantly greater illness severity during the acute infection phase. Differences in illness severity by serology group were also observed with the RRV, QF and serologically unconfirmed groups all reporting greater illness severity compared to the EBV group. In the multivariable analysis, lower mood disturbance scores in the acute illness were independently associated with older age. NPY genotype was not associated with either overall illness severity or mood disturbance in the acute infection phase.

**Table 2:** Univariate model for overall illness severity and mood disturbance endophenotype during the acute illness phase (baseline).

**PCs**	**Overall illness severity**		**Mood disturbance severity**	
**Predictors**	**Estimated mean difference (95% CI)**	***p*-value**	**Estimated mean difference (95% CI)**	***p*-value**
Female sex (vs. male)	0.27 (0.09, 0.46)	**0.01**	0.18 (−0.01, 0.37)	0.07
Age (per year)	−0.01 (−0.01, 0.00)	0.07	−0.01 (−0.02, −0.00)	**<0.001**
Serology (vs. EBV)		**0.01**		0.18
*RRV*	0.35 (0.06, 0.64)		0.27 (0.02, 0.52)	
*QF*	0.32 (0.01, 0.62)		0.22 (−0.09, 0.53)	
*Unconfirmed*	0.40 (0.16, 0.65)		0.14 (−0.15, 0.43)	
Diplotype (vs. HB1 homozygous)		0.50		0.77
*HB2 homozygous*	−0.10 (−0.36, 0.16)		−0.20 (−0.47, 0.06)	
*HB1/HB2 heterozygous*	0.11 (−0.12, 0.34)		0.13 (−0.22, 0.25)	
*HB1/HB3 heterozygous*	0.17 (−0.34, 0.68)		−0.00 (−0.52, 0.52)	
*HB1/HB4 heterozygous*	0.29 (−0.23, 0.81)		0.02 (−0.50, 0.55)	
*HB2/HB4 heterozygous*	−0.23 (−0.84, 0.37)		−0.23 (−0.85, 0.39)	
*HB3/HB2 heterozygous*	−0.22 (−0.83, 0.39)		−0.03 (−0.65, 0.59)	
*HB3/HB4 heterozygous*	−0.13 (−2.06, 1.81)		0.13 (−1.84, 2.10)	

*Data are shown as estimated mean differences (95% confidence interval). EBV, Epstein Barr Virus; QF, Q fever; RRV, Ross River Virus; HB1-4, NPY haplotype blocks (HB). Bold indicates statistically significant. PC, Principal components*.

### Predictors of Illness Duration

Participants with higher overall illness severity and mood disturbance severity PC scores in the acute phase of the illness had a delayed recovery for both the overall illness (HR = 0.39, 95% CI: 0.34–0.46, *p* < 0.001) and mood disturbance PCs (HR = 0.36, 95% CI: 0.30–0.42, *p* < 0.001), respectively. After accounting for other demographic and infection factors (age, sex, serology, NPY haplotype), prolonged illness at 6 months was positively associated with greater illness severity at baseline (*p* < 0.001) and RRV infection (*p* < 0.01) (Table 3). Being female and having greater baseline mood disturbance was associated with prolonged mood disturbance (Table 3). Logistic regression identified that the odds of the PIFS outcome was 2.4 times higher for each unit increase in illness severity score at baseline (i.e., the odds of PIFS increase with worse illness severity at baseline; Table 3). There were no associations with prolonged overall illness, prolonged mood disturbance, or PIFS with the NPY haplotypes, including when the previously reported low NPY-producing, highest-risk diplotype (HB1/HB1) was compared to the other diplotypes, no association was found (data not shown).

**Table 3:** Cox-regression model for time to recovery for overall illness severity and mood disturbance endophenotype.

**PCs**	**Overall illness severity**		**Mood disturbance**		**PIFS**	
**Predictors**	**Hazard ratio (95% CI)**	***p*-value**	**Hazard ratio (95% CI)**	***p*-value**	**Odds ratio (95% CI)**	***p*-value**
Female sex (vs. male)	0.80 (0.62, 1.04)	0.09	0.67 (0.54, 0.85)	**0.001**	0.72 (0.42, 1.25)	0.24
Age (per year)	1.01 (1.00, 1.01)	0.23	1.00 (0.10, 1.01)	0.30	1.01 (0.99, 1.03)	0.30
Serology (vs. EBV)		**0.02**		0.10		0.28
*RRV*	0.54 (0.37, 0.80)		0.65 (0.46, 0.93)		2.25 (0.96, 5.28)	
*QF*	0.73 (0.48, 1.09)		0.73 (0.51, 1.06)		2.10 (0.85, 5.20)	
*Unconfirmed*	0.80 (0.58, 1.11)		0.75 (0.55, 1.00)		1.69 (0.78, 3.66)	
Illness severity at baseline	0.40 (0.34, 0.46)	**<0.001**	Not included		2.24 (1.71, 2.94)	**<0.001**
Mood disturbance at baseline	Not included		0.36 (0.31, 0.43)	**<0.001**	Not included	
Diplotype (vs. HB1 homozygous)		0.42		0.23		0.77
*HB2 homozygous*	0.91 (0.64, 1.30)		0.86 (0.63, 1.18)		0.99 (0.46, 2.11)	
*HB1/HB2 heterozygous*	1.22 (0.90, 1.66)		0.85 (0.65, 1.12)		1.16 (0.60, 2.21)	
*HB1/HB3 heterozygous*	0.91 (0.45, 1.84)		1.27 (0.69, 2.33)		0.25 (0.29, 2.13)	
*HB1/HB4 heterozygous*	0.62 (0.28, 1.38)		0.48 (0.23, 1.01)		2.46 (0.68, 8.92)	
*HB2/HB4 heterozygous*	1.22 (0.60, 2.47)		1.42 (0.71, 2.84)		0.00 (0.00, 0.00)	
*HB3/HB2 heterozygous*	1.49 (0.71, 3.15)		1.40 (0.70, 2.82)		1.21 (0.22, 6.72)	
*HB3/HB4 heterozygous*	a		a		a	

*Hazard ratios (HR) are presented as the hazard of recovery (95% confidence interval for HR). A hazard ratio value of <1 indicates a reduced risk of being recovered at any given timepoint for a one-unit increase in the specific variable. The logistic regression model with PIFS as an outcome, is presented in the last column with odds ratios and 95% confidence intervals. EBV, Epstein Barr Virus; QF, Q fever; RRV, Ross River Virus; HB1-4, NPY haplotype blocks (HB); PC, Principal components. a. HB3/HB4 heterozygous dipolotype was included in the model but not reported due to the small sample size (n = 1). Bold indicates statistically significant*.

**Figure 1:**
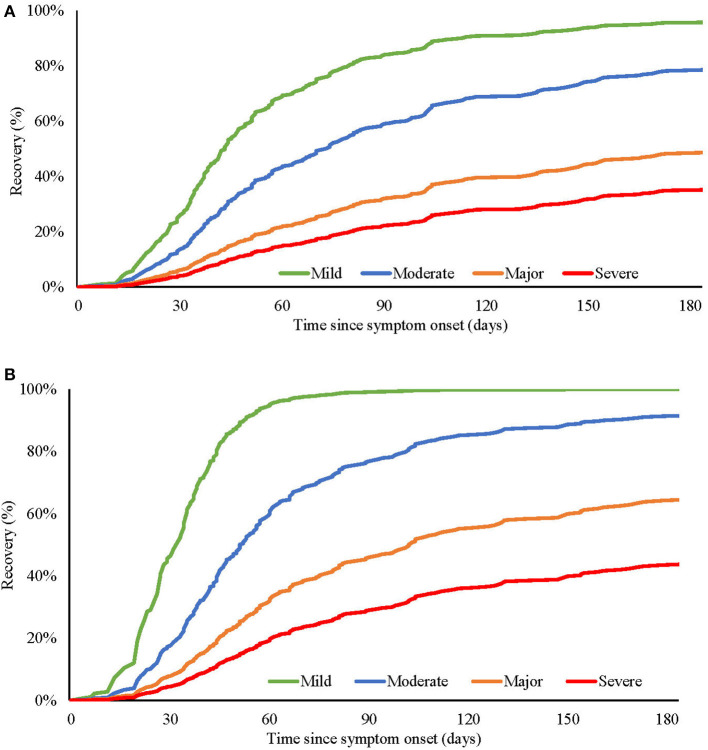
Predicted recovery of **(A)** overall illness severity and **(B)** mood disturbance severity, at different baseline levels (mild, moderate, major, and severe) of overall illness **(A)**, or mood disturbance **(B)**.

To better describe these severity-duration relationships, baseline severity scores were divided into quartiles. During the acute phase of the illness, the range of disability varied from a median of 15 days out of role and 4 days in bed for subjects in the “severe” group, compared to seven days out of role and 2 days in bed in the subject group with “mild” illness. The association with functional impairment was evident across illness severity quartiles, with 48% of “mild,” 46% of “moderate,” 70% of “major” and 72% of “severe” patients reporting having to cut down or stop their usual activities due to the acute illness. Similarly, as the severity of mood disturbance worsened, the proportion of patients with impacted social function increased: 27% (mild), 34% (moderate), 50% (major), 72% (severe). The longitudinal course to recovery in relation to the baseline illness severity quartiles (Figure 1A) revealed that by day 100, 86% of the “mild” severity subjects had recovered, as opposed to 23% of the “severe” subjects. Figure 1B shows the predicted recovery of mood disturbance similarly split by the severity of baseline mood disturbance, where by day 100, 99% of patients in the “mild” quartile had recovered compared to 31% in the “severe” quartile.

## Discussion

This study examined predictors of prolonged illness, prolonged mood disturbance, and PIFS following EBV, RRV, or QF infections. Illness characteristics (physical, behavioral, and mood symptoms) during the acute phase of the infection were typical of the acute sickness response, and continued in the prolonged illness in significant minority of subjects. Illness severity during the acute phase of the illness predicted the overall illness duration, including the PIFS outcome at 6 months. Similarly, greater mood disturbance during the acute infection predicted prolonged mood disturbance. Despite previous associations between genetic variations in NPY and mood disturbance (28), no association was confirmed in this cohort.

The case rate for PIFS (i.e., meeting the diagnostic criteria for CFS at 6 months) in this study was 19%. This case rate is consistent with previous reports from prospective cohort studies ranging between 10% and 35% (3). Although the pathogens in this study (EBV, RRV and QF) are typically self-limiting acute infections and are rarely associated with hospitalization or mortality (29–31), the subjects in DIOS were substantially affected by the acute illness. This is evidenced by the high number of “days out of role” and “days spent in bed” during the acute phase of the illness. Consistent with the severity of illness at baseline predicting PIFS, subjects who were later diagnosed with PIFS had a considerably higher number of days in bed at baseline compared to the cohort as a whole.

Compared to the EBV group, the other infection groups (RRV, QF, unconfirmed) and female gender were associated with higher overall illness severity during the acute phase of the illness. Broadly comparable findings have been reported previously by our group (14, 23). The RRV group was associated with a greater severity of musculoskeletal pain, consistent with the typical presentation of arthralgia and arthritis in this illness (1). Identifying those with severe initial illness is key since severity of the acute illness severity predicted both delayed recovery and PIFS caseness. This finding is consistent with previous reports from both our group and others whereby clinical features indicative of the severity of the acute infection predicted PIFS (1, 7). These findings also complement our previous reports, in which symptom severity during the acute illness phase, and not viral or immune markers predicted PIFS (1, 15, 18, 23). Subjects with RRV were more likely to have delayed recovery compared to those with EBV. Differences between infection groups have been observed in previous reports from the DIOS cohort, with subjects with confirmed RRV and QF having been reported to have lower probability of recovery from fatigue compared to EBV (23). This association may reflect the interaction between the pathogen and the host response, which underpin a spectrum of symptom manifestations during the acute illness and the stereotyped manifestations of acute sickness response (14). It should also be noted that nearly one-third of the cohort were defined as “unconfirmed” serology – whereby an acute infection was evident, but the pathogen was not serologically confirmed. This group had comparable illness severity indices and prolonged course including PIFS, consistent with the notion that a wide variety of acute infections may trigger the onset of PIFS – likely exemplified in long COVID (3).

Lower mood disturbance severity at baseline was independently predicted by older age. This was a somewhat unexpected finding since older age is typically associated with a greater risk of mood disturbance (23) and has also been consistently reported as a predictor for chronic fatigue following infection (9, 32). It should be noted that the DIOS cohort participants were relatively young overall with a median age of 32 years suggesting that mood disturbance in the context of acute infection may have different determinants than the prevalent counterparts of major depression and anxiety disorder occurring sporadically in the community. More severe mood disturbance during the acute infection predicted prolonged mood disturbance suggesting that post-infective mood disturbance share disease mechanisms with the acute sickness response. These findings are consistent with a single previous report (23). Female gender was also associated with delayed recovery from mood disturbance consistent with previous observations from DIOS in which females were over-represented in those with more severe mood disturbance (14). Some previous studies of delayed convalescence from acute infections have reported that premorbid psychiatric illness predicted post-infective major depression (6, 33), but not PIFS (1, 33).

As both the acute sickness response and CFS have strong genetic influences (34, 35), and given the links between severe acute sickness response to infection and PIFS, previous genetic association studies in DIOS have shown that both the acute phase severity and the prolonged illness duration are associated with functional SNPs in cytokine genes. The SNPs positively associated with illness severity included those in: immunological genes including the NLRP3 inflammasome (22), the pro-inflammatory cytokine genes [interleukin (IL)-1β, IL-6, tumor necrosis factor (TNF)-α, interferon (IFN)-γ] (14, 18) and the anti-inflammatory cytokine, IL-10 gene (18). In addition, functional SNPs in the gene encoding the purinergic P2X7 receptor, which is widely expressed in the immune system and brain, have also been associated with illness severity and duration (36). Additionally, individual symptom domains or endophenotypes of the illness complex have been differentially associated such as an IFN-γ SNP with fatigue, an IL-6 SNP with mood disturbance, and an IL-10 SNP with neurocognitive difficulties (14).

Genetic haplotype-driven variations in NPY expression have been previously shown to predict brain responses to stress and inversely correlate with trait anxiety (21, 28). Although the haplotype blocks derived from the DIOS population were concordant with previous reports (28), there was no association between NPY diplotypes and overall illness severity or mood disturbance severity either during the acute illness phase or prolonged illness. In particular, it was anticipated that the low NPY-producing diplotype (HB1/HB1) previously shown to confer high-risk for stress, anxiety, and low mood (21, 28) would be associated with mood disturbance during and after acute infection. This was not confirmed.

### Strengths and Limitations

DIOS was a well-characterized longitudinal cohort which rigorously applied a case definition of CFS that included comprehensive medical and psychiatric assessments, exclusionary laboratory investigations, and continuity of the prolonged fatigue state from the onset of the infective illness through to 6 months. The analysis presented here included a comprehensive investigation of the relationship between the acute illness characteristics and delayed recovery, including both a continuous outcome measure (duration of illness, duration of mood disturbance) and a categorical outcome (PIFS caseness). This report considered post-infective fatigue states resulting from three very different pathogens and a likely heterogenous “unconfirmed pathogen” group – but all triggered fatigue. The recruitment challenges in a large region of rural Australia and *via* general practice, resulted in delays between the initial onset of symptoms and study enrolment, which may have influenced the symptom reports at baseline.

## Conclusion

The severity of the acute infective illness is a key predictor of prolonged illness and PIFS, as well as post-infective mood disturbance across a range of infectious diseases. Early identification of this risk may allow early intervention and better outcomes.

## Data Availability Statement

The raw data supporting the conclusions of this article will be made available by the authors, without undue reservation.

## Ethics Statement

The studies involving human participants were reviewed and approved by Human Research Ethics Committee at University of New South Wales (HC190102, HC04257). The patients/participants provided their written informed consent to participate in this study.

## Author Contributions

AL and IH led the design and implementation of the DIOS. CS, HL, BV, and AL led the genetic associations analysis. CS, EC, and AL led the PCA and statistical analysis. CS led the manuscript preparation. All authors have contributed to the manuscript conception and design and approved the final manuscript.

## Funding

This work was supported in part by National Health and Medical Research Council of Australia (NHMRC) Project Grants [157052 and 157092], the Judith Jane Mason and Harold Stannet Williams Memorial Foundation (The Mason Foundation) Australia, and a Cooperative Research Agreement with the US Centers of Disease Control and Prevention [U50/CCU019851-01]. CS was supported by the Cancer Institute NSW Early Career Fellowship (2021/ECF1310). AL was supported by NHMRC Practitioner Fellowship (1041897).

## Conflict of Interest

The authors declare that the research was conducted in the absence of any commercial or financial relationships that could be construed as a potential conflict of interest.

## Publisher's Note

All claims expressed in this article are solely those of the authors and do not necessarily represent those of their affiliated organizations, or those of the publisher, the editors and the reviewers. Any product that may be evaluated in this article, or claim that may be made by its manufacturer, is not guaranteed or endorsed by the publisher.
