# Supplementary material for: Predictors of Chronic Fatigue Syndrome and Mood Disturbance After Acute Infection
Source: Front Neurol. 2022 Jul 25;13:935442. doi: 10.3389/fneur.2022.935442 (PMC9359311; doi:10.3389/fneur.2022.935442)
Supplement: Supplementary file 1 [file Table_1.pdf]

**Supplementary table S1.** SPHERE items contributing to overall illness severity PC, based on the sub-sample with time since symptom onset ≤42 days at intake (n=378).

| Item | Mean | SD | Extraction Communalities | Component Score Coefficient |
|---|---|---|---|---|
| *Illness severity* | | | | |
| Muscle pain after activity? | .80 | .842 | .366 | .076 |
| Needing to sleep longer? | 1.31 | .784 | .359 | .075 |
| Prolonged tiredness after activity? | 1.23 | .797 | .390 | .078 |
| Poor sleep? | .93 | .879 | .334 | .072 |
| Poor concentration? | .75 | .796 | .303 | .069 |
| Tired muscles after activity? | 1.09 | .840 | .343 | .073 |
| Feeling irritable or cranky? | .54 | .706 | .528 | .091 |
| Pains in your arms or legs? | .67 | .818 | .405 | .079 |
| Feeling nervous or tense? | .28 | .526 | .348 | .074 |
| Waking up tired? | 1.02 | .846 | .536 | .091 |
| Rapidly changing moods? | .56 | .752 | .507 | .089 |
| Arms or legs feeling heavy? | .65 | .767 | .445 | .083 |
| Back pain? | .50 | .726 | .377 | .077 |
| Joint pain? | .66 | .807 | .331 | .072 |
| Weak muscles? | .80 | .794 | .446 | .083 |
| Feeling frustrated? | .57 | .703 | .524 | .090 |
| Getting annoyed easily? | .69 | .751 | .551 | .093 |
| Everything getting on top of you? | .47 | .683 | .444 | .083 |
| Feeling tired after rest or relaxation? | .81 | .793 | .473 | .086 |

SD: standard deviation
