# Supplementary material for: Predictors of Chronic Fatigue Syndrome and Mood Disturbance After Acute Infection
Source: Front Neurol. 2022 Jul 25;13:935442. doi: 10.3389/fneur.2022.935442 (PMC9359311; doi:10.3389/fneur.2022.935442)
Supplement: Supplementary file 2 [file Table_2.pdf]

Supplementary table S2. SPHERE items contributing to mood disturbance PC scores, based on the sub-sample with time since symptom onset ≤42 days at intake (n=378).

Item	Mean	SD	Extraction Communalities	Component Score Coefficient
Mood disturbance				
Feeling irritable or cranky?	.54	.706	.552	.127
Feeling nervous or tense?	.28	.526	.458	.116
Waking up tried?	1.02	.846	.367	.104
Rapidly changing moods?	.56	.752	.590	.132
Feeling unhappy/depressed?	.47	.656	.393	.108
Feeling constantly under strain?	.47	.605	.372	.105
Feeling frustrated?	.57	.703	.655	.139
Getting annoyed easily?	.69	.751	.623	.135
Everything getting on top of you?	.47	.683	.693	.143
Being unable to overcome difficulties?	.26	.523	.394	.108
Feeling lost for the word?	.25	.536	.306	.095
Losing confidence?	.24	.510	.424	.112

SD: standard deviation
