# Supplementary material for: Predictors of Chronic Fatigue Syndrome and Mood Disturbance After Acute Infection
Source: Front Neurol. 2022 Jul 25;13:935442. doi: 10.3389/fneur.2022.935442 (PMC9359311; doi:10.3389/fneur.2022.935442)
Supplement: Supplementary file 3 [file Image_1.pdf]

Supplementary figure 1

(A)

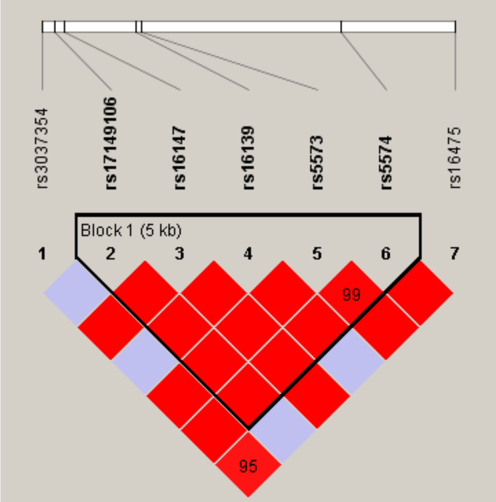

(B)

Haplotype	rs17149106 (2)	rs16147 (3)	rs16139 (4)	rs5573 (5)	rs5574 (6)	Frequency
HB1	G	G	A	A	T	0.479
HB2	G	A	A	G	C	0.459
HB3	T	A	G	G	C	0.030
HB4	G	G	A	A	C	0.029

Supplementary figure 1: Panel A. Haploview analysis of linkage disequilibrium in the NPY gene using seven marker SNPs. The colours represent the relative D'/LOD score where: bright red is D'=1, LOD $\geq$ 2, blue D'=1, LOD < 2. The D' values indicate linkage disequilibrium between each the selected SNPs. The 5-SNP haplotype block represents five variants with their relative frequencies in the study population listed in panel B.
